# Determinants of Job-Finding Intentions Among Young Adults from 11 European Countries

**DOI:** 10.1007/s11205-022-02941-6

**Published:** 2022-07-07

**Authors:** Francisco Simões, Jale Tosun, Antonella Rocca

**Affiliations:** 1 Instituto Universitário de Lisboa (ISCTE-IUL), CIS-IUL, Avª das Forças Armadas, 1649-026 Lisbon, Portugal; 2 Institute of Political Science, Heidelberg University, Bergheimer Str. 58, 69115 Heidelberg, Germany; 3 Department of Quantitative and Business Studies, University of Naples, “Parthenope”, 80132 Napoli, Italy

**Keywords:** Education, Mobility, Professional expectations, School-to-work transition, Skills

## Abstract

In this study we investigate the impact of individual, parental and social factors on young adults’ job-finding intentions across countries and overall. We test our hypotheses by conducting binary logistic regressions on the basis of an original dataset that comprises responses from more than 5200 young adults and their parents from 11 European countries. Our findings show that individual factors are most decisive in shaping the job-finding intentions of young adults. Most importantly, being enrolled in education does not prevent young adults from lowering their aspirations with respect to anticipated earnings or finding more sophisticated jobs. Social factors, in terms of being socially involved or having more friends in employment, lead to stronger mobility intentions as well as to stronger intentions to improve skills or develop new ones. At the country level, and compared to Italian participants, who go through a longer school-to-work transition, participants from Northern or Central European countries, as well as from the United Kingdom, show weaker mobility intentions. In many of these countries, young adults are also more willing than their Italian counterparts to lower income aspirations, while being less inclined to lower their aspirations in terms of finding more sophisticated jobs. In a last step, we reflect on education as a means of improving professional aspirations and the need to offer adequate on-the-ground services to help young people through the school-to-work transition, especially in Southern European countries.

**Supplementary Information:**

The online version contains supplementary material available at 10.1007/s11205-022-02941-6.

## Introduction

Youth employment has long been a central feature of the European Union’s (EU) policy agenda and especially since the economic crisis that unfolded after 2008. Of the policy measures adopted recently, the European Youth Guarantee, which addresses various aspects of the School-To-Work Transition (STWT), stands out as the flagship policy instrument. Its implementation has resulted in the adoption of a diverse set of policy instruments in the EU member states, including tools covering education and training, subsidized employment, and labor market services (Tosun, Arco-Tirado, et al., 2019; Tosun, Treib, et al., 2019). However, these policy instruments address only certain aspects of the challenges young adults face when searching for employment.

In advanced economies, numerous factors shape the subjective indicators of youth employment, such as job-finding intentions, which refer to the steps individuals are willing to take to find a job. At the individual level, education and vocational skills are important for explaining the likelihood of a young person to become (un)employed (see, e.g., Isengard, 2003). Parental scaffolding (mostly in the form of financial aid or the provision of housing), which can result in delays in the transition to adulthood (Swartz et al., 2011), or social networks, through friendships, are just a few examples of parental- or social-level factors that influence the educational and professional trajectories of young adults.

In light of this, we aim to explore how job-finding intentions are associated with individual, parental and social factors as well as how these intentions vary across European countries. We target three types of job-finding intentions: first, *mobility intentions*, which comprise different kinds of national and international spatial mobility (going, staying or returning) in order to improve job prospects (Frändberg, 2014; Maunaye, 2013); second, *qualification aspirations*, which refer to future-oriented cognitions involving alternative opportunities for improving skills or retraining (Rimkute et al., 2012); third, *professional aspirations*, which capture an individual’s desired occupation (Lee & Byun, 2019).

To address our goal, we draw on data from the project Cultural Pathways to Economic Self-Sufficiency and Entrepreneurship in Europe (CUPESSE). The resulting dataset comprises responses from more than 20,000 young adults (aged 18–35) from 11 European countries (Tosun, Arco-Tirado, et al., 2019; Tosun, Treib, et al., 2019). Out of these, more than 5200 responses from young adults and their parents form the empirical basis of this study.

The remainder of this article unfolds as follows. First, we briefly discuss the notion of STWT to contextualize the relevance of our research. Afterwards, we outline how different individual, parental and social factors are associated with job-finding intentions overall. Following this, we discuss why job finding intentions can differ between countries. Then, we provide details on the design of this study as well as on how we took measurements and conducted our analysis. Finally, we present our findings and discuss them.

## Conceptual Clarifications on School-to-Work Transition

The International Labor Organization defines the STWT as “the passage of a young person (aged 15–29 years) from the end of schooling to the first fixed-term or satisfactory employment” (Matsumoto & Elder, 2010). It consists of a sequence of transitions that vary across both individuals and countries (Brzinsky-Fay, 2007). This process occurs toward the end of adolescence and the beginning of adulthood, when young people go through an intricate process of identity formation (Masdonati et al., 2021). STWT processes are shaped by the socio-economic structures, institutional arrangements, and cultural patterns in place in a country, as well as by processes such as Europeanization or globalization (Walther, 2006).

STWT has become a prominent concept in both academic and public debates. Its relevance comes from evidence that a smooth STWT tackles labor market supply demands, ensures that human capital is not lost, and guarantees an independent lifestyle for younger generations (Brzinsky-Fay, 2014). Moreover, an effective STWT decreases the risk of the “work-experience trap”, which describes a situation in which “youngsters have an increasing level of education and, often, also enough general work experience, but firms want job-specific work experience and competences” (Pastore & Zimmermann, 2019, 374).

However, mounting evidence shows that a smooth STWT is becoming the exception; more often than not, it is characterized by individualization, fragmentation, and delays (Arnett, 2014; Brzinsky-Fay, 2007; Pastore & Zimmermann, 2019; Walther, 2006). This development is not a problem per se, and its roots can also be seen in social modernization processes such as extended periods of education, an increase in the participation levels of women in the labor market, and the opportunity to experiment with different career paths (Arnett, 2014; Masdonati et al., 2021; Walther, 2006).

Yet prolongment of the STWT process, the uncertainty associated with it, especially for individuals belonging to vulnerable groups (e.g., women, migrants or refugees; see Masdonati et al., 2021), and the consequent delayed entry into the labor market (Brzinsky-Fay, 2007) can have negative impacts on individuals. This is particularly true in countries with ‘closed’ labor markets that treat young adults as ‘outsiders’ who must compete for employment with ‘insiders’ due to a de-standardized STWT. More generally, STWT trajectories can affect young adults’ job-finding intentions (Cuconato, 2017), which then at the aggregate level are reflected in changes in the (un)employment indicators.

## Individual Factors that Influence Job-Finding intentions

Multiple individual factors shape young adults’ job-finding intentions. For instance, research has provided robust support that gender affects the intentions and actions of young adults. For example, young men are more likely to move within and outside the country in order to find a job (van Mol, 2016). Young women living in rural (Theodori & Theodori, 2015) or peri-urban areas and who come from multicultural or economically deprived areas (Maunaye, 2013) are less likely to consider moving an option in order to improve their career and employment prospects (Weiss et al., 2021).

Internal and external mobility predisposition is also a function of age. Overall, mobility intentions decrease as a person grows older (van Mol, 2016). The age-dependence of mobility intentions is reasonable considering that the transition to adulthood includes several milestones (Arnett, 2014). Moving out of the family home, leaving school, entering the labor market, or starting a family can thwart mobility intentions. The age trend overlaps with the evolution of educational and professional aspirations, which progress and become more stable after completing secondary school (Rimkute et al., 2012), although recent empirical studies have also shown that this tendency declines if young adults encounter significant adversity in their mid- or late-twenties (Almeida & Simões, 2020).

Educational attainment levels also shape job-finding intentions. Educated individuals display stronger mobility intentions (van Mol, 2016), especially those who do not feel pressured to move for economic reasons (Salamońska & Czeranowska, 2019). Individuals with lower school attainment or negative educational trajectories have lower prospects of attaining higher qualifications, especially when facing greater social and economic adversity before and after their transition to the labor market (Rimkute et al., 2012), such as in the case of young people who are Neither in Employment nor in Education and Training (NEETs) (Almeida & Simões, 2020).

Another factor influencing job finding intentions is income. Individuals who consume more are less likely to move abroad for employment-related reasons, unlike the unemployed (van Mol, 2016) or underemployed (Salamońska & Czeranowska, 2019), who can feel pressured to do so. Overall, financially deprived individuals have limited opportunities for experimenting with different types of work and are thus more likely to lower their professional aspirations to find a secure job, even when this comes at the expense of accepting offers that do not match their skills and interests (Kay et al., 2017).

## Parental Influence on Job-Finding Intentions

As Bourdieu and Passeron (2016) compellingly argue, parents’ financial, social and cultural resources play an important role in shaping the job-finding intentions of their children over their lifetimes, including in their transition to the labor market. Parental wealth facilitates youth migration, both within the country and abroad (Cairns, 2017). Affluent parents can facilitate the mobility of their children by providing critical material support or intangible resources such as broader social networks (Lee & Byun, 2019). Parents also shape their children’s mobility intentions through their own mobility behavior and perceptions of mobility, also known as ‘mobility capital’ (Hu & Cairns, 2017). This capital develops through life events, such as previous migration or divorce, or through positive family beliefs regarding migration, namely when family interactions are oriented towards the promotion of individuality over collectivism (Maunaye, 2013).

The parents’ education level and income status jointly influence their children’s professional aspirations and future qualifications. The former determines the children’s educational attainment and subsequent educational and occupational aspirations even more than household income does (Aina et al., 2015). This relationship can be explained through multiple pathways: Parents with higher educational attainment levels usually socialize their children into believing that success is rooted in meritocracy and the ability to control life circumstances (Cemalcilar et al., 2018; Kay et al., 2017). Such parents are usually also more involved in education and tend to value the role of education more highly (Rimkute et al., 2012), which ultimately boosts their children’s educational and professional aspirations. Moreover, the importance of attaining a certain professional status can be learned through the direct observation of role models when they exhibit professional behavior (Cemalcilar et al., 2018).

Parental expectations may also regulate children’s educational and professional aspirations through support enactment. If the amount of support provided by parents is seen by children as proportionate in relation to the expected personal outcomes of the transition to adulthood, this results in a securing effect. Parents supporting their children financially when they are enrolled in tertiary education is a good example of this. Conversely, disproportionate levels of parental support may convey messages of incompetence and failure, leading young people to lower their professional expectations, which can eventually result in downward social mobility (Manzoni, 2018).

## Social Influences on Job-Finding Intentions

The transition to adulthood offers structured opportunities for social participation through civic, political or social associations (Arnett, 2014). Children from families with high social capital benefit from this as they can access and use these existing social networks, which is one of the channels through which social class is reproduced (Bourdieu & Passeron, 2016).

The intergenerational transmission of social capital can have negative effects on mobility intentions. Greater levels of local involvement may deter persons from migrating to find work, particularly in rural areas (Theodori & Theodori, 2015). However, social capital is also a good proxy for openness to new education or work experience, inducing individuals, especially the most privileged ones, to foresee mobility as a chance to undertake new civic, political or social projects with a view to forming their own identity (Cairns, 2017). Persons eager to become part of social networks usually feel the need to strengthen their education and professional qualifications, believing these will help them to gain access to new or more prestigious groups. Therefore, it is conceivable that individuals who value social ties are also more likely to value education and career because these provide vehicles for strengthening social ties. This relationship has not attracted much attention in the literature yet.

Friendships can also shape job-seeking intentions. From the early twenties to the early thirties, friendships become less numerous, more stable and more intimate (Arnett, 2014). Friends are a relevant source of social comparison, which, in turn, affects the individuals’ achievement and happiness levels (Adler & Seligman, 2016). Moreover, individual perceptions of social inequality are predominantly determined by interpersonal comparisons rather than by income (Kelley & Evans, 2017). What has not been covered in the literature yet is how the career attainment of a young adult’s friends is associated with his/her job-finding intentions.

## Job-Finding Intentions across European Countries

Job-finding intentions vary considerably between European countries due to several cultural, policy-making and institutional factors (Brzinsky-Fay, 2014; Cuzzocrea, 2018).

From a cultural standpoint, meaning the dominant social values, collectivistic and individualistic orientations are particularly relevant for understanding job-finding intentions. In countries more oriented towards collectivistic values, young adults tend to rely more on informal social networks to shape up their job-finding intentions or to make concrete efforts to enter the labor market (Cuzzocrea, 2018). Consequently, loyalty to informal social groups, especially to families, dominates over personal merit and community problem-solving as ways of increasing social capital and improving job-finding prospects (Bello & Cuzzocrea, 2018; Simões & Rio, 2020). As a result, these cultural disparities are associated with more prolonged and complex transitions to the labor market in Southern European countries, which are often paired with more pessimistic expectations about entering the job market. There, independent adult life, in terms of leaving the parents’ house or seeking financial autonomy, begins well into the mid-thirties (e.g. Italy) (Cuzzocrea, 2018). This is much later than in the Northern and Central European countries (e.g. Denmark, Germany) included in our study (Carcillo et al., 2015).

Welfare, employment and education policy measures also contribute to distinguishing countries in terms of job-finding intentions. Welfare provision to young people ranges considerably across European countries—from the comprehensive safety net offered in Northern countries (e.g., Denmark) (Walther, 2006), to the high levels of informal social protection typical of Southern countries (e.g. Greece or Italy). The latter is due to both a lack of resources and the above-mentioned centrality of families in this transitional period (Bello & Cuzzocrea, 2018).

Employment systems also have an impact on how young people tailor their job-finding intentions. Northern countries implement on-the-ground employment policies to mitigate risks such as unemployment, precariousness or low-skilled jobs. Some states adopt a more liberal approach, exposing young people to these risks more often (e.g. United Kingdom). Others seek to coordinate vocational training tracks with immediate employability, especially across Central Europe. Yet another group of countries, typically those in Southern Europe, show unstructured approaches to the labor market inclusion of younger generations (Walther, 2006; Jagannathan, 2021).

These differences on youth employment policies are paralleled by distinctive education policies. Northern countries’ education systems are comprehensive, reducing streaming to a minimum and providing flexible training opportunities (Helms Jørgensen et al., 2019). This contrasts with the increasing number of private offers in countries such as the United Kingdom, the selective educational tracks in countries such as Germany, and the non-selective but still insufficient educational coverage in some Southern countries (e.g. Spain) (Walther, 2006). Overall, more nuanced welfare, employment and education policies create a safety net for young people, tailoring their job finding intentions in multiple ways. More structured policies in these areas increase short-term mobility for improving social and professional capital, raise educational expectations in general, and provide opportunities for accessing more complex and demanding jobs (Bello & Cuzzocrea, 2018; Carcillo et al., 2015; Helms Jørgensen et al., 2019).

Differences in institutional factors, in terms of governance views and traditions as well as policy implementation also affect job finding intentions across European countries. Specifically, policy design and implementation in EU countries in the realm of STWT are strongly affected by horizontal coordination (between different governance sectors) and vertical coordination (between priorities located at different decision-making levels—local/regional, national or European) (Tosun et al., 2019).

Horizontal coordination in the definition and implementation of STWT policies is frequently challenged by a dominant, bureaucratic culture of policy-making silos, with only a few interconnections between sectoral policies and a severe loss of efficiency (Marques, 2015). This lack of interconnection between vital sectors such as welfare, employment or education seems to be more challenging in some of the Southern countries covered in our study, such as Greece or Italy (Carcillo et al., 2015; Cuzzocrea, 2018). As a matter of fact, in some Northern countries, such as Denmark, and others not included in our research (e.g. Sweden), policy-design for younger generations results more often in integrated services deliverance (e.g. one-stop-shops), nurturing more informed job-finding intentions and therefore improving the efficiency of job-seeking and employability for young adults (Carcillo et al., 2015).

In turn, vertical coordination in the STWT domain encompasses well-known problems in translating broadband European policies, such as the Youth Guarantee, into meaningful policies and programs at the national or regional levels (Shore & Tosun, 2019). These problems translate into a shortage of on-the-ground resources, a lack of services or the autonomy to implement the policy measures (Shore & Tosun, 2019), or a mismatch between policy priorities and young people’s needs and expectations (Simões & Rio, 2020; Jagannathan, 2021). The challenges to vertical coordination in the STWT field are highlighted in the different Youth Guarantee national evaluation reports of different countries such as Germany (European Commission, 2020a), Hungary (European Commission, 2020b) or Spain (European Commission, 2020c). However, as we move from North to South, vertical STWT policy coordination seems more constrained, in part due to a shrinking number of on-the-ground institutional resources, especially of counseling services (Cuzzocrea, 2018). The shortage of these services especially in Southern countries complicates the development of realistic job-finding intentions among young people due to limited information about job opportunities (Carcillo et al., 2015), restricted access to adequate training (Almeida & Simões, 2020) or more difficult matching between personal qualifications and job offers (Kay et al., 2017).

## Present Study: A Comprehensive Investigation of Job-Finding Intentions

Which social (e.g. social involvement), parental (e.g. standard of living) or individual (e.g. age) factors affect young adults’ job-finding intentions across the different countries? This is our first research question, which we associate with four hypotheses that we derived on the basis of existing studies discussed above.

### H1:

Men as well as younger participants, the more educated ones, and those in employment or education will also score more highly on the indicators of mobility intentions. The same pattern will be found for qualifications and professional aspirations, with the exception that, in this case, women will present higher qualifications and professional aspirations than men.

### H2:

Participants whose parental situation is more positive in terms of stronger perceived influence on their children’s career and education, who are more satisfied with their financial situation, show greater educational levels, and are enrolled in education or are employed will display greater mobility intentions as well as higher qualifications and professional aspirations.

### H3:

Being more socially involved and having more friends in employment will be associated with greater mobility intentions as well as higher qualifications and professional aspirations.

### H4:

Overall, Northern and Central European countries are expected to present lower mobility intentions and qualifications aspirations, but higher professional aspirations compared to Southern countries, after controlling for individual, parental and social factors.

We consider this study to contribute to the literature by empirically assessing the extent to which factors covering several layers of reality (individual, parental and social levels) are associated with indicators of job-finding intentions across European countries. Our research perspective reflects, therefore, recent calls for developing and testing explanatory models that uncover the factors behind relevant subjective indicators that affect youth employability such as job-finding intentions (Masdonati et al., 2021). Our conceptual approach provides a useful basis for informing policies to be adopted in the aftermath of the COVID-19 pandemic, considering that young people have been affected very strongly due to the negative implications for education and training (International Labor Organization, 2020).

## Data and Methodology

In this section, we explain the database of this study, including the measurement of the key variables, and the analytical approach applied.

### Participants

This study uses data from the CUPESSE project, which surveyed respondents representing two generations of the same families, i.e., the children’s generation as the primary respondents and the parents’ generation as the secondary respondents. Another feature of this dataset is that it gathers data for respondents based in Austria, the Czech Republic, Denmark, Germany, Greece, Hungary, Italy, Spain, Switzerland, Turkey, and the United Kingdom (Tosun, Arco-Tirado, et al., 2019).

The youth questionnaire was fielded with the assistance of survey companies, which were asked to provide a probability sample of individuals aged 18–35 years who were representative of employment status (e.g., employed; self-employed; unemployed; in education/training), NUTS2 regions (which corresponds to the focal point of the EU’s regional policies), age group, education, and migration background/minority group membership. The parents’ survey was also conducted with the assistance of survey companies and using the contact information provided by the representatives of the children’s generation. Both surveys were fielded in 2016. The age range covered by this data is broader than with comparable research, which is the reason why we refer to “young adults” or “young people” rather than “youths”. This age range was selected in response to the empirical findings reported, for example, by Baranowska-Rataj et al. (2017) that young people are reaching the milestones of adulthood later than earlier generations did. Therefore, we wanted to capture potential delays in the transition to adulthood by collecting data from individuals covering a broader age range.

In terms of sample size, the minimum requirement per country was 1000 young adult respondents and 500 parents, with a reasonable proportion of fathers and mothers, which resulted in a dataset consisting of 20,008 observations. Of these, 5945 contained information on at least one parent. In cases where we had responses from both mothers and fathers, we deleted the information provided by the mothers and took only the information provided by the fathers into account, leaving us with 5445 observations for the parents. We did this to increase the share of the fathers’ responses relative to those given by the mothers. The share of responses by mothers’ is around 60 percent and by fathers around 40 percent.

### Data Preparation and Operationalization

The indicators of job-finding intentions that we included in this study are: moving within the same country (migrate internally); moving to a different country (migrate externally); improving skills; learning new skills; and lowering expectations concerning earnings or lowering expectations regarding professional conditions and responsibilities. The respondents could indicate their degree of willingness on a 3-point scale, ranging from “no” (1) to “maybe” (2) and “yes” (3). In the estimation of logistic models, we recoded these indicators of job-finding intentions as follows: 0 corresponds to the modalities “no” and “maybe” and 1 to “yes”. Table 2 in the Appendix describes in detail the outcome and explanatory variables. Table 1 presents the descriptive statistics for the whole sample. The corresponding detailed descriptive statistics by country are reported in Table 3 in the Appendix.

**Table 1:** Descriptive Statistics

Variable	Mean	SD	Min	Max
Migrate internally	0.43	0.49	0.28 (CZ)	0.59 (ES)
Migrate externally	0.31	0.46	0.13 (CH)	0.42 (IT)
Improve skills	0.78	0.41	0.47 (TK)	0.89 (ES)
Develop new skills	0.65	0.48	0.47 (TK)	0.74 (ES)
Lower exp. earnings	0.27	0.44	0.19 (HU)	0.48 (DK)
Lower exp. conditions	0.28	0.45	0.16 (HU)	0.49 (DK)
Female	0.55	0.50	0.50 (ES)	0.62 (DK)
Age	26.33	5.04	24.01 (TK)	28.05 (GR)
Education	4.57	1.76	3.74 (HU)	5.67 (CH)
*Professional condition*				
Employed	0.51	0.50	0.39 (TK)	0.71 (UK)
In education	0.28	0.45	0.17 (IT)	0.48 (CH)
Inactive	0.10	0.30	0.06 (DK-DE-GR)	0.19 (IT)
Unemployed	0.11	0.31	0.01 (CH)	0.22 (GR)
Satisfaction	2.46	0.87	2.02 (GR)	3.45 (CH)
Future standard P	3.45	1.00	2.69 (GR)	3.94 (TK)
Influence education P	2.42	0.62	2.05 (DK)	2.75 (ES)
Influence career P	1.98	0.68	1.64 (DK)	2.22 (HU)
Satisfaction P	2.63	0.83	1.93 (GR)	3.18 (DK)
Education P	3.61	1.91	1.71 (TK)	5.58 (CH)
*Professional condition P*				
Employed P	0.60	0.49	0.22 (TK)	0.78 (CH)
In education P	0.17	0.38	0.10 (CH)	0.32 (GR)
Inactive P	0.17	0.38	0.05 (CZ)	0.61 (TK)
Unemployed P	0.06	0.23	0.02 (AT-DK-CH-TK-UK)	0.11 (ES)
Social activity	1.01	1.19	0.36 (HU)	1.80 (CH)
Friends employed	3.17	0.99	2.32 (TK)	3.83 (DK-DE)

Responses given by parents refer to both mothers and fathers. Responses from mother = 3440 (approx. 60%) and from fathers = 2303 (approx. 40%)

**Table 2:** Description of the variables

Variable name	Question	Response categories
Migrate internally	What are you willing to do for a job/better job? Move within [country]	1: No; 2: Maybe; 3: Yes
Migrate externally	What are you willing to do for a job/better job? Move to a different country'	1: No; 2: Maybe; 3: Yes
Improve skills	What are you willing to do for a job/better job? Learn new skills (language, computer programs)	1: No; 2: Maybe; 3: Yes
Develop new skills	What are you willing to do for a job/better job? Learn completely new skills/retrain	1: No; 2: Maybe; 3: Yes
Lower expectations, earnings	What are you willing to do for a job/better job? Lower expectations regarding earnings	1: No; 2: Maybe; 3: Yes
Lower expectations, conditions	What are you willing to do for a job/better job? Lower expectations regarding conditions/responsibility	1: No; 2: Maybe; 3: Yes
Female	Are you female or male?	Female = 1 male = 0
Age	How old are you?	In years
Education	What is the highest level of education you achieved?	1: less than lower secondary (ES-ISCED I); 2: lower secondary (ES-ISCED II); 3: lower tier upper secondary (ES-ISCED IIIb); 4: upper tier upper secondary (ES-ISCED IIIa); 5: advanced vocational (ES-ISCED IV); 6: lower tertiary education (ES-ISCED VI); 7: higher tertiary education (ES-ISCED VII)
Professional condition	And which of these descriptions best describes your situation in the last month? Please select only one option	1: working; 2: in education/training; 3: not working; 4: unemployed
Satisfaction	Thinking about your own financial situation, how satisfied are you right now?	1: Very dissatisfied; 2: Rather dissatisfied; 3: Rather satisfied; 4: Very satisfied
Future standard P	Question asked to parents: Thinking about how your child's standard of living will be like in the future…	1: Much worse than mine; 2: Worse; 3: Similar; 4: Better; 5: Much better than mine
Influence education P	Question asked to parents: When you think about the influence you had on your child’s life path until today, what would you say about the amount of influence you have had on his/her education?	1: no influence; 2 some influence; 3: a lot of influence
Influence career P	Question asked to parents: When you think about the influence you had on your child’s life path until today, what would you say about the amount of influence you have had on his/her career?	1: no influence; 2 some influence; 3: a lot of influence
Satisfaction P	Question asked to parents: How satisfied are you with your own financial situation?	1: Very dissatisfied; 2: Rather dissatisfied; 3: Rather satisfied; 4: Very satisfied
Education P	Question asked to parents: What is the highest level of education you achieved?	1: less than lower secondary (ES-ISCED I); 2: lower secondary (ES-ISCED II); 3: lower tier upper secondary (ES-ISCED IIIb); 4: upper tier upper secondary (ES-ISCED IIIa); 5: advanced vocational (ES-ISCED IV); 6: lower tertiary education (ES-ISCED VI); 7: higher tertiary education (ES-ISCED VII)
Professional condition P	Question asked to parents: And which of these descriptions best describes your situation in the last month? Please select only one option	1: working; 2: in education/training; 3: not working; 4: unemployed
Social activity	During a normal week roughly how many hours are you voluntarily involved in organizations such as charities, environmental organizations, sport clubs or cultural organizations?	0: 0 h; 1: Less than 1 h; 2: 1–3 h; 3: 4–7 h; 5: 8 + hours
Friends employed	Thinking about your friends, how many of them are employed?	1: None of them; 2: A few of them; 3: Some of them; 4: Most of them; 5: All of them

**Table 3:**
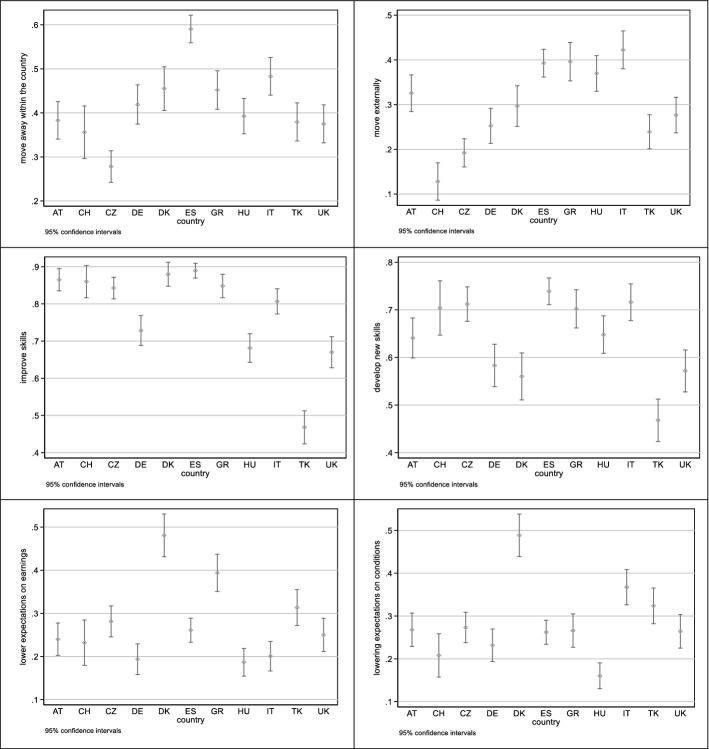
Mean values for the job-finding intentions and confidence levels

The first set of explanatory variables is measured at the individual level for the young respondents and includes information on gender (*Female*), age (*Age*), and educational attainment (*Education*), as well as on the *Professional Condition* (Employed, in education/training, inactive, or unemployed) and the degree to which they are satisfied with their financial situation (*Satisfaction*).

Regarding parental factors, we included the same set of variables for mothers and fathers, covering their expectations regarding the future standard of living of their children (*Future Standard P*), their perceptions of how much they have influenced their children’s education and career-related decisions (*Influence Education P* and *Influence Career P*), their satisfaction with their financial situation (Satisfaction P), their educational attainment levels (Education P), and their employment status (*Professional Condition P*).

Concerning social factors, the models include information on how involved the young adults are in social activities, such as charities (Social Activity), and on the number of friends in employment they have (*Friends Employed*).

**Fig. 1:**
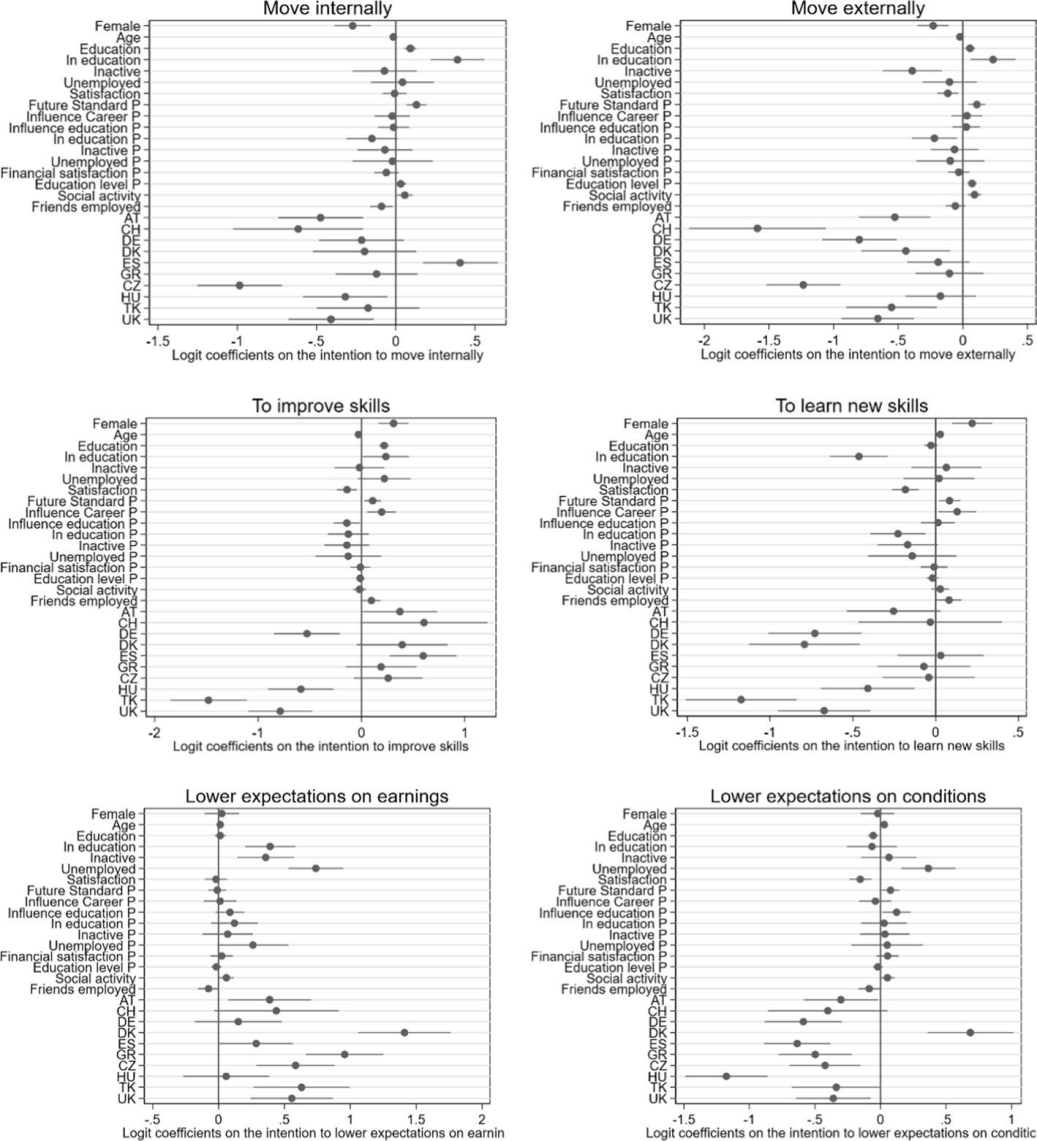
Logit coefficients for dummy variables measuring the effect of countries on job-finding intentions

Figure 1 shows the differences in job-finding behaviors across countries. The shares of young people declaring their willingness to move within or outside the country are higher in the Mediterranean countries of Spain (59% within and 39% outside the country), Italy (48% and 42%, respectively) and Greece (45% and 40%), as well as in Denmark (46% and 30%), while it is lowest for young people living in the Czech Republic (28% and 19%) and Switzerland (36% and 13%). The corresponding shares for the whole sample are, respectively, 43% and 31%. Improving skills is an intention stated especially by those living in Central and Southern Europe (over 80% in all these countries, with the exception of Germany, where it reaches 73%), while in Turkey, the United Kingdom and Hungary this intention is not very spread (with shares, respectively, of 47%, 67% and 68%). Young people from Turkey and the United Kingdom, together with their Danish and German peers, also appear less likely to want to develop new skills. Finally, almost one in two Danish young people is willing to lower expectations regarding earnings and professional condition, while in Hungary fewer than two in ten are.

Table 4 in the Appendix shows the indexes of association between the variables measuring the different job-finding intentions and the other covariates. For variables based on a nominal scale (female and main professional condition), we report the chi-square measure of association, while for those on the ordinal scale, we calculated the Kendall rank correlation, which allows us to detect the direction of the correlation. The corresponding statistical tests of significance show that, with the exception of lower expectations regarding conditions and responsibilities, all the job-finding intentions are strongly connected to gender and the professional condition of both young individuals and their parents.

**Table 4:** Kendall Rank Correlations between job-finding intentions and the other covariates

Covariates	Move internally	Move externally	Improve skills	Develop new skills	Lower exp. earnings	Lower exp. conditions
*Chi-square*						
Female	62.42^***^	67.59^***^	26.74^***^	14.79^***^	15.22^***^	3.84
Professional condition	98.24^***^	96.44^***^	38.51^***^	64.38^***^	132.96^***^	57.23^***^
Professional condition P	15.05^**^	59.31^***^	66.68^***^	24.94^***^	20.72^***^	7.35
*Kendall Rank correlations*						
Age	− 0.0367^***^	− 0.0673^***^	0.0022	0.0732^***^	− 0.0020	0.0185^*^
Education	0.0595^***^	0.0544^***^	0.0769^***^	0.0138	0.0052	− 0.0239^**^
Satisfaction	− 0.0122	− 0.0279^***^	− 0.0087	− 0.0378^***^	− 0.0139	− 0.0324^***^
Future standard P	0.0192^*^	− 0.0057	− 0.0009	− 0.0023	− 0.0016	0.0042
Influence education P	0.0240^**^	0.0322^***^	0.0313^***^	0.0179^**^	− 0.0030	− 0.0189^**^
Influence career P	0.0255^***^	0.0331^***^	− 0.0000	0.0102	0.0029	− 0.0131
Satisfaction P	0.0005	− 0.0088	0.0056	− 0.0190^**^	0.0037	0.0150
Education P	0.0290^***^	0.0756^***^	0.0507^***^	− 0.0117	0.0044	− 0.0098
Social activity	0.0380^***^	0.0638^***^	0.0400^***^	0.0193^**^	0.0337^***^	0.0375^***^
Friends employed	− 0.0458^***^	− 0.0359^***^	0.0199^***^	0.0380^***^	− 0.0445^***^	− 0.0263^***^
Move internally						
Move externally	0.2917^***^	–				
Improve skills	0.0872^***^	0.0927^***^	–			
Develop new skills	0.0637^***^	0.0551^***^	0.1794^***^	–		
Lower exp. Earnings	0.0758^***^	0.0270^***^	0.0437^***^	0.0776^***^	–	
Lower exp. conditions	0.0528^***^	0.0170	0.0423^***^	0.0865^***^	0.3371^***^	–

*p < 0.10; **p < 0.05; ***p < 0.01

The willingness to take action to find a job increases when young people are unsatisfied with their financial condition or when parents perceive themselves as having a greater influence on their children’s education and career. We also find trends regarding an active social life: Young adults widely involved in social engagement (charities, environmental organizations and sport activities) show stronger rates across the different, subjective job-finding intentions.

The intention to move within or outside the country to find a job is higher for younger individuals and for those with a higher level of education. The latter are also more oriented to improving their skills, but not to lowering expectations regarding their professional condition.

Focusing only on the job-finding intentions, the Kendall correlations are all positive and statistically significant at 0.01, showing that those who want to find a job are often prone to taking action/are likely to take action in different ways. In particular, there is a moderate correlation between the respondents’ willingness to migrate internally and to migrate externally (0.29). We can also see that there is a moderate correlation between the respondents’ willingness to improve their skills and to learn new skills (0.18). Lastly, the responses to lowering expectations regarding earnings or conditions and responsibilities also show a moderate degree of correlation (0.34).

### Analytical Strategy

We ran binary logistic regression models for each job-intention behavior, including the respondents from all countries, and controlled for country effects by computing robust standard errors and including country dummies (Table 5 in the Appendix). We used Italy as the reference category, as previous studies have identified the Italian school-to-work transition as the longest in Europe (Pastore et al., 2021).

**Table 5:** Average marginal effects for models with country dummies

	Move internally	Move externally	Improve skills	Develop new skills	Lower exp. earnings	Lower exp. conditions
Female	− 0.06^***^	− 0.05^***^	0.05^***^	0.05^***^	0.01	− 0.00
	[− 0.09, − 0.04]	[− 0.07, − 0.02]	[0.02,0.07]	[0.02,0.07]	[− 0.02,0.03]	[− 0.03,0.02]
Age	− 0.00^*^	− 0.00^**^	− 0.00^***^	0.01^***^	0.00	0.01^***^
	[− 0.01, − 0.00]	[− 0.01, − 0.00]	[− 0.01, − 0.00]	[0.00,0.01]	[− 0.00,0.01]	[0.00,0.01]
Education	0.02^***^	0.01^*^	0.03^***^	− 0.01	0.00	− 0.01^**^
	[0.01,0.03]	[0.00,0.02]	[0.03,0.04]	[− 0.01,0.00]	[− 0.01,0.01]	[− 0.02, − 0.00]
Employed	0.00	0.00	0.00	0.00	0.00	0.00
	[0.00,0.00]	[0.00,0.00]	[0.00,0.00]	[0.00,0.00]	[0.00,0.00]	[0.00,0.00]
In education	0.09^***^	0.06^**^	0.03^*^	− 0.10^***^	0.07^***^	− 0.01
	[0.05,0.13]	[0.02,0.09]	[0.00,0.07]	[− 0.14, − 0.07]	[0.04,0.11]	[− 0.05,0.02]
Inactive	− 0.01	− 0.07^**^	0.00	0.02	0.07^**^	0.01
	[− 0.06,0.04]	[− 0.11, − 0.03]	[− 0.04,0.04]	[− 0.03,0.06]	[0.02,0.11]	[− 0.03,0.06]
Unemployed	0.01	− 0.02	0.03	0.00	0.15^***^	0.07^***^
	[− 0.04,0.05]	[− 0.06,0.02]	[− 0.01,0.07]	[− 0.04,0.05]	[0.10,0.19]	[0.03,0.12]
Satisfaction	− 0.00	− 0.02^**^	− 0.02^**^	− 0.04^***^	− 0.00	− 0.03^***^
	[− 0.02,0.02]	[− 0.04, − 0.01]	[− 0.04, − 0.01]	[− 0.06, − 0.02]	[− 0.02,0.01]	[− 0.05, − 0.01]
Future Standard P	0.03^***^	0.02^**^	0.02^**^	0.02^**^	− 0.00	0.01^*^
	[0.02,0.04]	[0.01,0.04]	[0.00,0.03]	[0.00,0.03]	[− 0.01,0.01]	[0.00,0.03]
Influence	− 0.01	0.01	0.03^**^	0.03^*^	0.00	− 0.01
Career P	[− 0.03,0.02]	[− 0.02,0.03]	[0.01,0.05]	[0.00,0.05]	[− 0.02,0.03]	[− 0.03,0.02]
Influence	− 0.00	0.00	− 0.02^*^	0.00	0.02	0.02^*^
Education P	[− 0.03,0.02]	[− 0.02,0.03]	[− 0.04, − 0.00]	[− 0.02,0.02]	[− 0.00,0.04]	[0.00,0.04]
Financial	− 0.01	− 0.01	− 0.00	− 0.00	0.00	0.01
Satisfaction P	[− 0.03,0.00]	[− 0.02,0.01]	[− 0.02,0.01]	[− 0.02,0.02]	[− 0.01,0.02]	[− 0.01,0.03]
Education level P	0.01	0.01^***^	− 0.00	− 0.00	− 0.00	− 0.00
	[− 0.00,0.02]	[0.01,0.02]	[− 0.01,0.00]	[− 0.01,0.00]	[− 0.01,0.00]	[− 0.01,0.00]
Employed P	0.00	0.00	0.00	0.00	0.00	0.00
	[0.00,0.00]	[0.00,0.00]	[0.00,0.00]	[0.00,0.00]	[0.00,0.00]	[0.00,0.00]
In education P	− 0.03	− 0.04^*^	− 0.02	− 0.05^**^	0.02	0.01
	[− 0.07,0.00]	[− 0.08, − 0.01]	[− 0.05,0.01]	[− 0.09, − 0.01]	[− 0.01,0.06]	[− 0.03,0.04]
Inactive P	− 0.02	− 0.01	− 0.02	− 0.03	0.01	0.01
	[− 0.06,0.02]	[− 0.05,0.02]	[− 0.05,0.01]	[− 0.07,0.00]	[− 0.02,0.05]	[− 0.03,0.04]
Unemployed P	− 0.00	− 0.02	− 0.02	− 0.03	0.05	0.01
	[− 0.06,0.05]	[− 0.08,0.03]	[− 0.07,0.03]	[− 0.09,0.03]	[− 0.01,0.10]	[− 0.04,0.06]
Social activity	0.01^*^	0.02^***^	− 0.00	0.01	0.01^*^	0.01^*^
	[0.00,0.03]	[0.01,0.03]	[− 0.01,0.01]	[− 0.00,0.02]	[0.00,0.02]	[0.00,0.02]
Friends employed	− 0.02^**^	− 0.01	0.01^*^	0.02^*^	− 0.01	− 0.02^*^
	[− 0.04, − 0.01]	[− 0.03,0.00]	[0.00,0.03]	[0.00,0.03]	[− 0.03,0.00]	[− 0.03, − 0.00]
Denmark	− 0.04	− 0.09^**^	0.06	− 0.17^***^	0.26^***^	0.14^***^
	[− 0.12,0.03]	[− 0.16, − 0.02]	[− 0.01,0.13]	[− 0.24, − 0.10]	[0.19,0.32]	[0.08,0.20]
Austria	− 0.11^***^	− 0.11^***^	0.06^*^	− 0.06	0.06^*^	− 0.06^*^
	[− 0.17, − 0.05]	[− 0.17, − 0.06]	[0.00,0.11]	[− 0.12,0.00]	[0.00,0.12]	[− 0.12, − 0.01]
Switzerland	− 0.11^*^	− 0.32^***^	0.09	− 0.00	0.09^*^	− 0.07
	[− 0.20, − 0.01]	[− 0.43, − 0.21]	[− 0.00,0.18]	[− 0.09,0.09]	[0.00,0.18]	[− 0.16,0.02]
Germany	− 0.05	− 0.17^***^	− 0.08^**^	− 0.16^***^	0.02	− 0.12^***^
	[− 0.11,0.01]	[− 0.23, − 0.11]	[− 0.12, − 0.03]	[− 0.22, − 0.10]	[− 0.04,0.08]	[− 0.17, − 0.06]
United Kingdom	− 0.09^**^	− 0.14^***^	− 0.12^***^	− 0.14^***^	0.09^**^	− 0.07^*^
	[− 0.15, − 0.03]	[− 0.20, − 0.08]	[− 0.16, − 0.07]	[− 0.20, − 0.09]	[0.04,0.15]	[− 0.13, − 0.02]
Spain	0.10^***^	− 0.04	0.10^***^	0.00	0.04	− 0.12^***^
	[0.04,0.15]	[− 0.09,0.01]	[0.05,0.15]	[− 0.05,0.06]	[− 0.01,0.10]	[− 0.17, − 0.08]
Greece	− 0.03	− 0.03	0.03	− 0.02	0.17^***^	− 0.10^***^
	[− 0.09,0.03]	[− 0.08,0.03]	[− 0.02,0.08]	[− 0.08,0.04]	[0.12,0.22]	[− 0.15, − 0.04]
Czech Republic	− 0.22^***^	− 0.26^***^	0.04	− 0.01	0.10^***^	− 0.08^**^
	[− 0.28, − 0.16]	[− 0.32, − 0.20]	[− 0.01,0.10]	[− 0.07,0.05]	[0.04,0.16]	[− 0.13, − 0.03]
Hungary	− 0.06^*^	− 0.03	− 0.08^***^	− 0.08^**^	0.02	− 0.19^***^
	[− 0.12, − 0.00]	[− 0.09,0.03]	[− 0.13, − 0.04]	[− 0.14, − 0.02]	[− 0.04,0.08]	[− 0.25, − 0.13]
Turkey	− 0.03	− 0.05	− 0.20^***^	− 0.22^***^	0.11^**^	− 0.06
	[− 0.10,0.05]	[− 0.13,0.02]	[− 0.25, − 0.14]	[− 0.29, − 0.15]	[0.05,0.18]	[− 0.13,0.00]
*N*	5277	5272	5281	5275	5276	5269
*AIC*	10,669.46	11,003.73	6011.79	497.88	10,911.42	10,941.55

95% confidence intervals in brackets; *p < 0.05, **p < 0.01, ***p < 0.001; covariates indicated with the letter “*P*” refer to responses given by the parent

While the outcome variables of interest are coded as ordinal responses (“yes”, “maybe” and “no”), model specifications with ordered logistic regression analyses violated the assumption of parallel slopes. Consequently, we fitted logistic regressions with robust standard errors. Table 5 in the Appendix shows the coefficients of the model, indicating the change in the probability that a respondent replies with ‘yes’ as well as their confidence intervals (CI). These results are graphically reported in Fig. 1.

## Results

In this section, we first discuss the individual, parental and social factors that shape job-finding intentions. Then, we concentrate on the differences in the individuals’ job-finding intentions across the countries studied.

### Individual Factors and Job-Finding Intentions

Regarding the connections between *individual factors and job-finding indicators*, we found that internal (− 0.27; CI: − 0.39; − 0.16) and external mobility (− 0.23; CI: − 0.35; − 0.11) intentions were lower among women and older participants (− 0.02; for both mobility types; CI respectively of − 0.03; − 0.00 and − 0.04; 0.04). Those who were less satisfied with their financial situation showed lower external migration intentions (− 0.12; CI: − 0.20; − 0.04). Conversely, internal (0.39; CI: 0.22; 0.56) and external (0.23, CI: 0.05; 0.40) mobility intentions were stronger among those who are still enrolled in education.

Moreover, women were more willing to both improve (0.31 CI: 0.16; 0.45) and develop new skills (0.22 CI: 0.10; 0.34). Seemingly the participants with a higher level of education also exhibited a significant intention to improve skills (0.22 CI: 0.17; 0.27). In addition, the participants who were still enrolled in education showed a positive intention to improve skills (0.23 CI: 0.01; 0.45), but a negative propension to develop new skills (− 0.47 CI: − 0.64; − 0.29). Also, as the participants became older, their intention to improve skills declined (− 0.03 CI: − 0.05; − 0.01), though they were significantly more willing to develop new skills (0.03 CI: 0.01; 0.04). A lower level of satisfaction with one’s financial situation overlapped significantly with weaker intentions of improving (− 0.14 CI: − 0.24; − 0.05) or developing (− 0.18; CI: 0.26; − 0.10) new skills.

Finally, participants who were enrolled in education (0.39 CI: 0.20; 0.58), inactive (0.36 CI: 0.14; 0.57) or unemployed (0.74 CI: 0.53; 0.94) were all willing to lower earnings expectations. Likewise, the more educated participants (− 0.06 CI: − 0.10; − 0.02) as well as those who were less satisfied with their financial situation (− 0.15 CI: − 0.24; − 0.07) were less willing to lower their aspirations regarding professional conditions and responsibilities, in contrast to those who were unemployed (0.36 CI: 0.16; 0.56).

### Parental Factors and Job-Finding Intentions

The associations between *family-level factors and indicators of job-finding intentions* were less recurrent. Still, we found that internal (0.13; CI: 0.07; 0.19) and external (0.11; 0.04; 0.17) mobility intentions increased when parents expected the future living standard of their children to be higher. The same trend was found for both internal (0.03 CI: − 0.00;0.06) and external mobility (0.07 CI: 0.03; 0.10) when parents showed a higher level of education.

Interestingly, if parents expected their children’s future living standard to be high, this increased the odds of the latter being willing to improve (0.11; CI: 0.03; 0.19) or develop (0.09; CI: 0.02; 0.15) new skills to find a job. The same pattern was found for the association between the parents’ perceived influence on their children’s career decisions, with a similar result for both improving skills intentions (0.20; CI: 0.06; 0.33) and for developing new skills (0.13 CI: 0.01; 0.25). Parental factors only marginally influenced professional aspirations. Parents having higher expectations regarding their children’s living standard (0.08; CI: 0.01; 0.14) and a stronger perceived influence on educational decisions (0.12; CI: 0.02; 0.23) were two factors associated with lowering expectations of conditions and responsibilities.

### Social Factors and Job-Finding Intentions

Remarkably, *social factors and job-finding intentions* were often linked, although the magnitude of the associations was small. A higher involvement in charities, environmental organizations and sports activities significantly increases intentions of moving within (0.06; CI: 0.01; 0.11) or outside the country (0.09; CI: 0.04; 0.14), while having more friends in employment reduces internal mobility intentions (− 0.09; CI: − 0.16; − 0.02).

Moreover, having more friends in employment contributed to increasing the willingness to improve (0.09; CI: 0.01; 0.18) or develop (0.08; CI: 0.00; 0.15) new skills. In addition, greater enrollment in civic or environmental organizations and sports activities was connected to stronger and identical odds of lowering expectations regarding earnings and job conditions and responsibilities (respectively, 0.05 and 0.06; CI: 0.00; 0.11).

### Cross-Country Differences in Job-Finding Intentions

After accounting for all individual, parental and social factors, we found that in general, all countries, when compared to the reference country (Italy), displayed lower mobility intentions. This was particularly evident for Northern and Central European countries and the United Kingdom, and the trend reached its peak in the cases of the Czech Republic for internal mobility (− 0.99; CI: − 1.25; − 0.72) and of Switzerland for external mobility (− 1.59; CI: − 2.12; − 1.06). However, Greek participants, unlike their Spanish counterparts (0.40 CI: 0.17;0.64), did not show significant intentions to move within or outside the country when compared to Italian participants.

Our findings for educational aspirations are less straightforward. In general, participants are less willing to improve skills in different latitudes of the European continent when compared to Italian participants. This is true for Central European countries such as Germany (− 0.53; CI: − 0.85; − 0.21) or Hungary (− 0.58 CI − 0.90; − 0.27), the United Kingdom (− 0.79; CI: − 1.; 10–0.48), and even Southeastern countries such as Turkey (− 1.48; CI: − 1.85; − 1.11). Denmark, Austria, Switzerland and Spain, however, show positive intentions. Aside, we found a generalized negative intention across countries for acquiring new skills; this was particularly evident in Northern (e.g. Denmark: − 0.79; CI: − 1.13; − 0.46), Central (e.g., Germany: − 0.73; CI: − 1.01; − 0.45), and Southeastern countries (e.g. Turkey: − 1.17; CI: − 1.51; − 0.84).

Finally, regarding professional aspirations, we found a reasonably consistent trend across countries for lowering earnings expectations, when compared to Italy, that peaked in the case of Denmark (1.41; CI: 1.06; 1.76). Interestingly, we found the opposite trend across many of the countries covered in our study, regarding lowering professional expectations. In other words, compared to Italian participants, participants from several countries were less inclined to lower their expectations of their future professional condition, whether that was the case of Central European countries such as Germany (− 0.64; CI: − 0.89; − 0.38) or countries in the South of the continent such as Spain (− 0.64; CI: − 0.89; − 0.38). Only the Danish participants were more inclined to lower their expectations regarding professional conditions than the Italian ones were (0.69; CI: 0.36; 1.01).

## Discussion

In our study, we assessed how individual, parental and social factors are associated with subjective job-finding indicators. We also tested how these intentions vary across countries, after controlling for those same factors. We reached four main findings.

First, regarding internal and external migration intentions, our findings uphold prior evidence that women face greater familial and cultural barriers to mobility (Maunaye, 2013; Theodori & Theodori, 2015). As we expected, mobility propension decreases with age and with lower satisfaction with one’s financial situation. Moreover, we also found that young adults still enrolled in education exhibit stronger mobility intentions. These results are coherent with the fact that younger generations build upon their cultural (Simões et al., 2021; Van Mol, 2016) and economic resources for mobility purposes (Salamońska & Czeranowska, 2019) but that these intentions decline as time goes by (Van Mol, 2016).

Second, women as well as more educated participants showed firmer intentions of improving or developing their skills. This finding is not surprising, considering that women are more academically minded (Farrugia, 2016) and that those accumulating greater cultural capital are more open to expanding their knowledge (Rimkute et al., 2012). It is interesting to note, however, that those still enrolled in education are interested in improving their skills, instead of developing new ones. This result comes as no surprise, because those still attending school envisage skills specialization as a leeway to facilitate their inclusion in the labor market. As these participants are also younger, it is reasonable to accept that they might also be unaware of the labour market barriers and demands (Simões et al., 2017), including requirements such as the need to diversify skills (Kay et al., 2017). This may result not only from the lack of work experience, but also from limited access to appropriate career guidance services (Shore & Tosun, 2019).

In turn, those more highly educated are also older and certainly have done some work experience that has helped them to understand the importance of developing new sets of skills. Incidentally, it is concerning to find that the belief in the existence of social upward mobility effects that result from education, a core element of the European social contract, seems to be eroding among those still attending school. Our results demonstrate that these participants significantly lowered their expectations regarding future earnings—much, albeit not to the same degree, as participants who were unemployed or inactive did (Kay et al., 2017). While these participants might not have been prepared by on-the-ground employment services for the specific features of contemporary STWT processes, they seem to know well that uncertainity dominates that particular life period and that it entails financial costs (Arnett, 2014).

Overall, parental factors had a limited impact in terms of scope and magnitude. We believe there are three reasons for this. To begin with, a great deal of the parental impact is related to socialization during childhood and adolescence (Cemalcilar et al., 2018). Through socialization in early years, parents pass on financial, social and cultural resources (Bourdieu & Passeron, 2016), specific beliefs (e.g. views on mobility), or access to broader social networks (Lee & Byun, 2019) that come to be part of the individual repertoire and patrimony. Therefore, parental influences are, to a certain extent, already reflected in individual factors that we included in the analyses. Aside, parents’ perceptions are distal factors vis-à-vis their children’s job-finding intentions when compared to enacted behaviors, such as material support (e.g., co-housing) (Manzoni, 2018). Finally, in the transition to adulthood, parents’ perceptions become less pivotal in forming their children’s future-oriented cognitions (Arnett, 2014). A good illustration of this interpretation is that overall, parental factors were barely connected to indicators of qualifications and professional aspirations, further showing that their role becomes less relevant to job-finding intentions as time goes by.

Third, we also found significant connections between social factors and job-finding intentions in general. These associations were, however, limited in magnitude, but still deserve some attention. Greater openness to social involvement comes with greater flexibility in terms of job-finding intentions, namely in terms of mobility. In our opinion, the social participation element reflects a wider identity formation process in which being more involved socially leads to greater openness to new experiences that are strongly connected to mobility (Cairns, 2017). In addition, it is important to note that reporting a higher number of friends in employment was associated with stronger qualifications aspirations. Social comparison plays an important role in the transition to adulthood in regulating decisions and positive feelings (Kay & Evans, 2017). Friendships become more selective, stronger, and can have a role model effect on younger adults’ decisions, including in educational decisions, as horizontal relationships become pivotal in identity formation and development throughout the twenties and thirties (Arnett, 2014).

Fourth, when compared to Italy, the European country with longer STWT processes (Pastore et al., 2021), we found that internal and external mobility intentions within the country and abroad are often significantly lower, with a few exceptions among other countries located toward the South (e.g. Spain). Across many of the Northern and Central European countries included in our analysis, it seems therefore that mobility is less relevant in increasing employability prospects. More favorable socioeconomic conditions in those countries—including state-provided social protection during the STWT (Helms Jorgensen et al., 2019; Walther, 2006), shorter and less erratic transitions (Pastore et al., 2021), and more robust institutional support (Schoon & Heckhausen, 2019)—certainly decrease the need to use mobility as a springboard for finding a job.

In terms of qualifications aspirations, however, it is important to note that compared to Italians, there was a generalized negative intention across countries for developing new skills. This seems to translate to different intra-country trends. In part, this result may reflect young adults’ challenges to access training and retraining, in countries such as Turkey. This may be due to a lack of comprehensive coverage of educational packages as well as limitations to matching young people’s needs with the available training packages (Shore & Tosun, 2019; Simões & Rio, 2020; Jagannathan, 2021). It is interesting to note, nevertheless, that Spanish and Greek participants do not differ from Italian ones in this respect. In other cases, however, this result certainly translates into a more secure STWT, especially in countries with significant on-the-ground vocational education systems, with a strong focus on immediate employability, as is the case in Germany (Walther, 2006).

Moreover, we found a reasonably consistent trend across countries of participants lowering earnings expectations as compared to Italian participants. Our finding further illustrates that younger generations across Europe are aware of the above-mentioned uncertainty of this transitional period, irrespective of the differences in duration that these processes can take from country to country (Brzinsky-Fay, 2014; Masdonati et al., 2021). Moreover, an increasingly fragmented STWT overlaps with a complex process of individual identity formation. This convergence between structural and individual challenges can strengthen feelings of pessimism and fear about the financial future that are recurrent among emergent adults (Arnett, 2014). In any case, this type of reasoning is, nevertheless, plausible considering that these participants were surveyed after having lived through the 2008 economic crisis.

## Limitations

Our study addresses job-finding intentions instead of enacted behaviors. These intentions were assessed using Likert scales, rather than the usually dichotomous (Yes/No) approach (Carling & Schewel, 2018). Furthermore, we know from research that intentions often translate into actual behavior (van Mol, 2016). Nevertheless, the well-known gap between intentions and concrete actions also applies to this research. The assessment of potential behavior can nevertheless inform policymakers by helping them to act by anticipation.

In terms of empirical testing, we must concede that our cross-sectional design provides a limited basis for a rigorous assessment of hypotheses.

Finally, parents were involved according to their availability, leading to a distinct difference in the participation of mothers and fathers. This enabled us to aggregate their views, which ensured the answers were more meaningful. Future research needs to ensure the simultaneous participation of mothers and fathers, to balance their view.

## Conclusion

Our study offers four main insights. First, individual factors significantly affect job-finding intentions among young people, across countries and overall. Second, we have contributed to the literature by showing that positive conditions associated with education do not prevent young adults from lowering their expectations. This erosion of education as a means to improve aspirations has important implications for the design of policies that aim to promote the employment of young adults. Third, our results indicate that the role of social factors in particular must be considered in order to develop a more complete understanding of the STWT, as social relationships greatly influence self-assessment in this developmental period. Finally, our results illustrate a general awareness among young people of the complexity of decision-making during the STWT. Adequate on-the-ground services are necessary to help young people to refine their job-finding intentions and to properly match their desires with available opportunities in the job market.

### Electronic supplementary material

Below is the link to the electronic supplementary material.

Supplementary file1 (DOCX 61 kb)

## Appendix

See Tables 2, 3, 4, 5.
