# Supplementary material for: Determinants of Job-Finding Intentions Among Young Adults from 11 European Countries
Source: Soc Indic Res. 2022 Jul 7;164(2):623–48. doi: 10.1007/s11205-022-02941-6 (PMC9261251; doi:10.1007/s11205-022-02941-6)
Supplement: Supplementary file 1 — Supplementary file1 (DOCX 61 kb) [file 11205_2022_2941_MOESM1_ESM.docx]

**Appendix**

Table A1: Description of the variables

| **Variable Name** | **Question** | **Response categories** |
| --- | --- | --- |
|  |  |  |
| Migrate internally | What are you willing to do for a job/better job? Move within [country] | 1: No; 2: Maybe; 3: Yes |
| Migrate externally | What are you willing to do for a job/better job? Move to a different country' | 1: No; 2: Maybe; 3: Yes |
| Improve skills | What are you willing to do for a job/better job? Learn new skills (language, computer programs). | 1: No; 2: Maybe; 3: Yes |
| Develop new skills | What are you willing to do for a job/better job? Learn completely new skills/retrain. | 1: No; 2: Maybe; 3: Yes |
| Lower expectations,  earnings | What are you willing to do for a job/better job? Lower expectations regarding earnings | 1: No; 2: Maybe; 3: Yes |
| Lower expectations,  conditions | What are you willing to do for a job/better job? Lower expectations regarding conditions/responsibility | 1: No; 2: Maybe; 3: Yes |
| Female | Are you female or male? | Female=1 male=0 |
| Age | How old are you? | In years |
| Education | What is the highest level of education you achieved? | 1: less than lower secondary (ES-ISCED I); 2: lower secondary (ES-ISCED II); 3: lower tier upper secondary (ES-ISCED IIIb); 4: upper tier upper secondary (ES-ISCED IIIa); 5: advanced vocational (ES-ISCED IV); 6: lower tertiary education (ES-ISCED VI); 7: higher tertiary education (ES-ISCED VII) |
| Professional condition | And which of these descriptions best describes your situation in the last month? Please select only one option. | 1: working; 2: in education/training; 3: not working; 4: unemployed |
| Satisfaction | Thinking about your own financial situation, how satisfied are you right now? | 1: Very dissatisfied; 2: Rather dissatisfied; 3: Rather satisfied; 4: Very satisfied |
| Future standard P | Question asked to parents: Thinking about how your child's standard of living will be like in the future… | 1: Much worse than mine; 2: Worse; 3: Similar; 4: Better; 5: Much better than mine |
| Influence education P | Question asked to parents: When you think about the influence you had on your child’s life path until today, what would you say  about the amount of influence you have had on his/her education? | 1: no influence; 2 some influence; 3: a lot of influence |
| Influence career P | Question asked to parents: When you think about the influence you had on your child’s life path until today, what would you say  about the amount of influence you have had on his/her career? | 1: no influence; 2 some influence; 3: a lot of influence |
| Satisfaction P | Question asked to parents: How satisfied are you with your own financial situation? | 1: Very dissatisfied; 2: Rather dissatisfied; 3: Rather satisfied; 4: Very satisfied |
| Education P | Question asked to parents: What is the highest level of education you achieved? | 1: less than lower secondary (ES-ISCED I); 2: lower secondary (ES-ISCED II); 3: lower tier upper secondary (ES-ISCED IIIb); 4: upper tier upper secondary (ES-ISCED IIIa); 5: advanced vocational (ES-ISCED IV); 6: lower tertiary education (ES-ISCED VI); 7: higher tertiary education (ES-ISCED VII) |
| Professional condition P | Question asked to parents: And which of these descriptions best describes your situation in the last month? Please select only one option. | 1: working; 2: in education/training; 3: not working; 4: unemployed |
| Social activity | During a normal week roughly how many hours are you voluntarily involved in organizations such as  charities, environmental organizations, sport clubs or cultural organizations? | 0: 0 hours; 1: Less than 1 hour; 2: 1-3 hours; 3: 4-7 hours; 5: 8+ hours |
| Friends employed | Thinking about your friends, how many of them are employed? | 1: None of them; 2: A few of them; 3: Some of them; 4: Most of them; 5: All of them |

Table A2: Mean values for the job-finding intentions and confidence levels

|  |  |
| --- | --- |
|  |  |
|  |  |

Table A3: Kendall Rank Correlations between job-finding intentions and the other covariates

| **Covariates** | **Move internally** | **Move externally** | **Improve skills** | **Develop new skills** | **Lower exp. earnings** | **Lower exp. conditions** |
| --- | --- | --- | --- | --- | --- | --- |
| *Chi-square* |  |  |  |  |  |  |
| Female | 62.42^***^ | 67.59^***^ | 26.74^***^ | 14.79^***^ | 15.22^***^ | 3.84 |
| Professional condition | 98.24^***^ | 96.44^***^ | 38.51^***^ | 64.38^***^ | 132.96^***^ | 57.23^***^ |
| Professional condition P | 15.05^**^ | 59.31^***^ | 66.68^***^ | 24.94^***^ | 20.72^***^ | 7.35 |
| *Kendall Rank correlations* | |  |  |  |  |  |
| Age | -0.0367^***^ | -0.0673^***^ | 0.0022 | 0.0732^***^ | -0.0020 | 0.0185^*^ |
| Education | 0.0595^***^ | 0.0544^***^ | 0.0769^***^ | 0.0138 | 0.0052 | -0.0239^**^ |
| Satisfaction | -0.0122 | -0.0279^***^ | -0.0087 | -0.0378^***^ | -0.0139 | -0.0324^***^ |
| Future standard P | 0.0192^*^ | -0.0057 | -0.0009 | -0.0023 | -0.0016 | 0.0042 |
| Influence education P | 0.0240^**^ | 0.0322^***^ | 0.0313^***^ | 0.0179^**^ | -0.0030 | -0.0189^**^ |
| Influence career P | 0.0255^***^ | 0.0331^***^ | -0.0000 | 0.0102 | 0.0029 | -0.0131 |
| Satisfaction P | 0.0005 | -0.0088 | 0.0056 | -0.0190^**^ | 0.0037 | 0.0150 |
| Education P | 0.0290^***^ | 0.0756^***^ | 0.0507^***^ | -0.0117 | 0.0044 | -0.0098 |
| Social activity | 0.0380^***^ | 0.0638^***^ | 0.0400^***^ | 0.0193^**^ | 0.0337^***^ | 0.0375^***^ |
| Friends employed | -0.0458^***^ | -0.0359^***^ | 0.0199^***^ | 0.0380^***^ | -0.0445^***^ | -0.0263^***^ |
| Move internally |  |  |  |  |  |  |
| Move externally | 0.2917^***^ | - |  |  |  |  |
| Improve skills | 0.0872^***^ | 0.0927^***^ | - |  |  |  |
| Develop new skills | 0.0637^***^ | 0.0551^***^ | 0.1794^***^ | - |  |  |
| Lower exp. Earnings | 0.0758^***^ | 0.0270^***^ | 0.0437^***^ | 0.0776^***^ | - |  |
| Lower exp. conditions | 0.0528^***^ | 0.0170 | 0.0423^***^ | 0.0865^***^ | 0.3371^***^ | - |

* p<0.10; ** p<0.05; *** p<0.01.

Table A4: Average Marginal Effects for Models with Country Dummies

|  | **Move internally** | **Move externally** | **Improve skills** | **Develop new skills** | **Lower exp. earnings** | **Lower exp. conditions** |
| --- | --- | --- | --- | --- | --- | --- |
| Female | -0.06^***^ | -0.05^***^ | 0.05^***^ | 0.05^***^ | 0.01 | -0.00 |
|  | [-0.09,-0.04] | [-0.07,-0.02] | [0.02,0.07] | [0.02,0.07] | [-0.02,0.03] | [-0.03,0.02] |
|  |  |  |  |  |  |  |
| Age | -0.00^*^ | -0.00^**^ | -0.00^***^ | 0.01^***^ | 0.00 | 0.01^***^ |
|  | [-0.01,-0.00] | [-0.01,-0.00] | [-0.01,-0.00] | [0.00,0.01] | [-0.00,0.01] | [0.00,0.01] |
|  |  |  |  |  |  |  |
| Education | 0.02^***^ | 0.01^*^ | 0.03^***^ | -0.01 | 0.00 | -0.01^**^ |
|  | [0.01,0.03] | [0.00,0.02] | [0.03,0.04] | [-0.01,0.00] | [-0.01,0.01] | [-0.02,-0.00] |
|  |  |  |  |  |  |  |
| Employed | 0.00 | 0.00 | 0.00 | 0.00 | 0.00 | 0.00 |
|  | [0.00,0.00] | [0.00,0.00] | [0.00,0.00] | [0.00,0.00] | [0.00,0.00] | [0.00,0.00] |
|  |  |  |  |  |  |  |
| In education | 0.09^***^ | 0.06^**^ | 0.03^*^ | -0.10^***^ | 0.07^***^ | -0.01 |
|  | [0.05,0.13] | [0.02,0.09] | [0.00,0.07] | [-0.14,-0.07] | [0.04,0.11] | [-0.05,0.02] |
|  |  |  |  |  |  |  |
| Inactive | -0.01 | -0.07^**^ | 0.00 | 0.02 | 0.07^**^ | 0.01 |
|  | [-0.06,0.04] | [-0.11,-0.03] | [-0.04,0.04] | [-0.03,0.06] | [0.02,0.11] | [-0.03,0.06] |
|  |  |  |  |  |  |  |
| Unemployed | 0.01 | -0.02 | 0.03 | 0.00 | 0.15^***^ | 0.07^***^ |
|  | [-0.04,0.05] | [-0.06,0.02] | [-0.01,0.07] | [-0.04,0.05] | [0.10,0.19] | [0.03,0.12] |
|  |  |  |  |  |  |  |
| Satisfaction | -0.00 | -0.02^**^ | -0.02^**^ | -0.04^***^ | -0.00 | -0.03^***^ |
|  | [-0.02,0.02] | [-0.04,-0.01] | [-0.04,-0.01] | [-0.06,-0.02] | [-0.02,0.01] | [-0.05,-0.01] |
|  |  |  |  |  |  |  |
| Future Standard P | 0.03^***^ | 0.02^**^ | 0.02^**^ | 0.02^**^ | -0.00 | 0.01^*^ |
|  | [0.02,0.04] | [0.01,0.04] | [0.00,0.03] | [0.00,0.03] | [-0.01,0.01] | [0.00,0.03] |
|  |  |  |  |  |  |  |
| Influence | -0.01 | 0.01 | 0.03^**^ | 0.03^*^ | 0.00 | -0.01 |
| Career P | [-0.03,0.02] | [-0.02,0.03] | [0.01,0.05] | [0.00,0.05] | [-0.02,0.03] | [-0.03,0.02] |
|  |  |  |  |  |  |  |
| Influence | -0.00 | 0.00 | -0.02^*^ | 0.00 | 0.02 | 0.02^*^ |
| Education P | [-0.03,0.02] | [-0.02,0.03] | [-0.04,-0.00] | [-0.02,0.02] | [-0.00,0.04] | [0.00,0.04] |
|  |  |  |  |  |  |  |
| Financial | -0.01 | -0.01 | -0.00 | -0.00 | 0.00 | 0.01 |
| Satisfaction P | [-0.03,0.00] | [-0.02,0.01] | [-0.02,0.01] | [-0.02,0.02] | [-0.01,0.02] | [-0.01,0.03] |
|  |  |  |  |  |  |  |
| Education level P | 0.01 | 0.01^***^ | -0.00 | -0.00 | -0.00 | -0.00 |
|  | [-0.00,0.02] | [0.01,0.02] | [-0.01,0.00] | [-0.01,0.00] | [-0.01,0.00] | [-0.01,0.00] |
|  |  |  |  |  |  |  |
| Employed P | 0.00 | 0.00 | 0.00 | 0.00 | 0.00 | 0.00 |
|  | [0.00,0.00] | [0.00,0.00] | [0.00,0.00] | [0.00,0.00] | [0.00,0.00] | [0.00,0.00] |
|  |  |  |  |  |  |  |
| In education P | -0.03 | -0.04^*^ | -0.02 | -0.05^**^ | 0.02 | 0.01 |
|  | [-0.07,0.00] | [-0.08,-0.01] | [-0.05,0.01] | [-0.09,-0.01] | [-0.01,0.06] | [-0.03,0.04] |
|  |  |  |  |  |  |  |
| Inactive P | -0.02 | -0.01 | -0.02 | -0.03 | 0.01 | 0.01 |
|  | [-0.06,0.02] | [-0.05,0.02] | [-0.05,0.01] | [-0.07,0.00] | [-0.02,0.05] | [-0.03,0.04] |
|  |  |  |  |  |  |  |
| Unemployed P | -0.00 | -0.02 | -0.02 | -0.03 | 0.05 | 0.01 |
|  | [-0.06,0.05] | [-0.08,0.03] | [-0.07,0.03] | [-0.09,0.03] | [-0.01,0.10] | [-0.04,0.06] |
|  |  |  |  |  |  |  |
| Social activity | 0.01^*^ | 0.02^***^ | -0.00 | 0.01 | 0.01^*^ | 0.01^*^ |
|  | [0.00,0.03] | [0.01,0.03] | [-0.01,0.01] | [-0.00,0.02] | [0.00,0.02] | [0.00,0.02] |
|  |  |  |  |  |  |  |
| Friends employed | -0.02^**^ | -0.01 | 0.01^*^ | 0.02^*^ | -0.01 | -0.02^*^ |
|  | [-0.04,-0.01] | [-0.03,0.00] | [0.00,0.03] | [0.00,0.03] | [-0.03,0.00] | [-0.03,-0.00] |
|  |  |  |  |  |  |  |
| Denmark | -0.04 | -0.09^**^ | 0.06 | -0.17^***^ | 0.26^***^ | 0.14^***^ |
|  | [-0.12,0.03] | [-0.16,-0.02] | [-0.01,0.13] | [-0.24,-0.10] | [0.19,0.32] | [0.08,0.20] |
|  |  |  |  |  |  |  |
| Austria | -0.11^***^ | -0.11^***^ | 0.06^*^ | -0.06 | 0.06^*^ | -0.06^*^ |
|  | [-0.17,-0.05] | [-0.17,-0.06] | [0.00,0.11] | [-0.12,0.00] | [0.00,0.12] | [-0.12,-0.01] |
|  |  |  |  |  |  |  |
| Switzerland | -0.11^*^ | -0.32^***^ | 0.09 | -0.00 | 0.09^*^ | -0.07 |
|  | [-0.20,-0.01] | [-0.43,-0.21] | [-0.00,0.18] | [-0.09,0.09] | [0.00,0.18] | [-0.16,0.02] |
|  |  |  |  |  |  |  |
| Germany | -0.05 | -0.17^***^ | -0.08^**^ | -0.16^***^ | 0.02 | -0.12^***^ |
|  | [-0.11,0.01] | [-0.23,-0.11] | [-0.12,-0.03] | [-0.22,-0.10] | [-0.04,0.08] | [-0.17,-0.06] |
|  |  |  |  |  |  |  |
| United Kingdom | -0.09^**^ | -0.14^***^ | -0.12^***^ | -0.14^***^ | 0.09^**^ | -0.07^*^ |
|  | [-0.15,-0.03] | [-0.20,-0.08] | [-0.16,-0.07] | [-0.20,-0.09] | [0.04,0.15] | [-0.13,-0.02] |
|  |  |  |  |  |  |  |
| Spain | 0.10^***^ | -0.04 | 0.10^***^ | 0.00 | 0.04 | -0.12^***^ |
|  | [0.04,0.15] | [-0.09,0.01] | [0.05,0.15] | [-0.05,0.06] | [-0.01,0.10] | [-0.17,-0.08] |
|  |  |  |  |  |  |  |
| Greece | -0.03 | -0.03 | 0.03 | -0.02 | 0.17^***^ | -0.10^***^ |
|  | [-0.09,0.03] | [-0.08,0.03] | [-0.02,0.08] | [-0.08,0.04] | [0.12,0.22] | [-0.15,-0.04] |
|  |  |  |  |  |  |  |
| Czech Republic | -0.22^***^ | -0.26^***^ | 0.04 | -0.01 | 0.10^***^ | -0.08^**^ |
|  | [-0.28,-0.16] | [-0.32,-0.20] | [-0.01,0.10] | [-0.07,0.05] | [0.04,0.16] | [-0.13,-0.03] |
|  |  |  |  |  |  |  |
| Hungary | -0.06^*^ | -0.03 | -0.08^***^ | -0.08^**^ | 0.02 | -0.19^***^ |
|  | [-0.12,-0.00] | [-0.09,0.03] | [-0.13,-0.04] | [-0.14,-0.02] | [-0.04,0.08] | [-0.25,-0.13] |
|  |  |  |  |  |  |  |
| Turkey | -0.03 | -0.05 | -0.20^***^ | -0.22^***^ | 0.11^**^ | -0.06 |
|  | [-0.10,0.05] | [-0.13,0.02] | [-0.25,-0.14] | [-0.29,-0.15] | [0.05,0.18] | [-0.13,0.00] |
| *N* | 5277 | 5272 | 5281 | 5275 | 5276 | 5269 |
| *AIC* | 10669.46 | 11003.73 | 6011.79 | 497.88 | 10911.42 | 10941.55 |

Notes: 95% confidence intervals in brackets; ^*^ *p* < 0.05, ^**^ *p* < 0.01, ^***^ *p* < 0.001; covariates indicated with the letter “P” refer to responses given by the parent.
